# Compressibility and Rheology of Clay Tailings: Effects of Sodium Polyacrylate in Presence of Divalent Cations

**DOI:** 10.3390/polym17141903

**Published:** 2025-07-09

**Authors:** Steven Nieto, Eder Piceros, Yanko Castañeda, Pedro Robles, Williams Leiva, Gonzalo R. Quezada, Ricardo I. Jeldres

**Affiliations:** 1 Water Research Center for Agriculture and Mining (CHRIAM), Victoria 1295, Concepción 4030000, Chile; yeison.nieto.mejia@ua.cl; 2 Facultad de Ingeniería y Arquitectura, Universidad Arturo Prat, Iquique 1100000, Chile; 3 Departamento de Ingeniería Química y Procesos de Minerales, Facultad de Ingeniería, Universidad de Antofagasta, Antofagasta 1240000, Chile; yanko.castaneda.tapia@ua.cl; 4 Escuela de Ingeniería Química, Pontificia Universidad Católica de Valparaiso, Valparaíso 2340000, Chile; pedro.robles@pucv.cl; 5 Facultad de Ingeniería, Universidad San Sebastián, Sede Concepción, Concepción 4030000, Chile; williams.leiva@uss.cl; 6 Departamento de Ingeniería de Procesos y Bioproductos, Facultad de Ingeniería, Universidad del Bío-Bío, Av. Collao 1202, Concepción 4030000, Chile; grquezada@ubiobio.cl; 7 Advanced Mininig Technology Center (AMTC), Universidad de Antofagasta, Antofagasta 1240000, Chile

**Keywords:** compressibility, rheology, clay tailings, sodium polyacrylate, divalent cations

## Abstract

Increasing water scarcity in arid regions has prompted the mining industry to develop strategies to maximize water recovery and reuse, especially in tailings treatment processes. In this context, the present investigation evaluated the effects of sodium polyacrylate (NaPA) on the compressibility and viscoelasticity of clayey tailings in the presence of hard water containing calcium and magnesium. To this end, clayey slurries were analyzed using rheological tests (rheograms and oscillatory viscoelasticity), zeta potential measurements, and compressibility tests using batch centrifugation. The yield stress was determined using the Herschel–Bulkley model, while the compressive yield stress (Py(Φ)) was calculated as a key indicator to characterize the degree of sediment consolidation. The results showed that NaPA, due to its anionic nature and high degree of ionization at pH 8, induces effective particle dispersion by increasing electrostatic repulsion and decreasing the interaction force between particles, which reduces both rheological parameters and compressive yield stress. For the 70/30 quartz/kaolin mixture, the yield stress decreased from 70.54 to 61.64 Pa in CaCl_2_ and from 57.51 to 52.95 Pa in MgCl_2_ in the presence of NaPA. It was also observed that suspensions in the presence of magnesium ions presented greater compressibility than those with calcium, attributable to the greater hydration radius of magnesium (10.8 Å), which favors less dense and more easily deformable network structures. Furthermore, a higher proportion of kaolin in the mixture resulted in higher yield stresses, a product of the clay’s laminar structure, colloidal size, and high surface area, both in the absence and presence of NaPA. Overall, the results show that incorporating NaPA significantly improves the compressibility and rheology of clayey tailings in hard water, offering a promising alternative for optimizing water recovery and improving tailings management efficiency in the context of water restrictions.

## 1. Introduction

Water use is essential for mining operations, as it enables the efficient extraction of valuable minerals. However, in arid regions where this resource is limited, the mining industry is forced to implement strategies aimed at its recovery, replacement, and reuse [1], promoting sustainability and environmental responsibility along with improved operational efficiency.

Water recovery in mining is primarily achieved through tailings thickening stages, where the tailings from the froth flotation process are treated. In this operation, the tailings are mixed with high-molecular-weight flocculants that adhere to the particles through various mechanisms, such as electrostatic patching, charge neutralization, and/or polymeric bridges, generating aggregates that settle rapidly. This process produces an overflow of clarified water, which is reusable in upstream processes, and a thickened sludge at the bottom, which is transported to tailings storage facilities (TSFs) [2].

The compressive yield strength is a key parameter for understanding and optimizing tailings sedimentation and disposal processes, which describes a sediment’s ability to withstand external loads before undergoing significant consolidation. This parameter is critical in tailings management, as understanding it allows for improved water recovery efficiency, optimized sediment behavior in thickeners, and guaranteed physical stability in storage tanks. As the compressive stress increases, sediment compaction becomes more difficult, leading to greater water retention in its internal structure. Conversely, low values favor the expulsion of interstitial water, thus facilitating more efficient consolidation. This consolidation phenomenon is directly related to greater water recovery through its release from within the slurry.

Understanding and controlling this parameter also allows for precise adjustments in the dosage of reagents, such as flocculants or rheology modifiers. Properly regulating reagent management can improve the structure of the formed sediment, increase the efficiency of the thickening process, and reduce the amount of water retained in the sludge [3,4,5,6]. Furthermore, compressive yield stress plays a crucial role in the geotechnical stability of tailings dams. Properly compacted sediment generates lower pore pressures and reduces water mobility within the dam, which helps to strengthen the structural integrity of retaining walls and mitigate risks such as liquefaction or tailings slides [7,8,9,10].

In physicochemical terms, the compressibility of sediments also depends on the composition of the process water. Cases of special interest are the corresponding studies in waters of low metallurgical quality or high ion concentrations. For example, Nasser and James [11] analyzed the compressive yield strength of kaolin sediments by varying the sodium chloride (NaCl) concentration and the pH. Their results indicated that increased salinity generates higher compressibility at lower compressive yield strength due to the face-to-face interactions of kaolin. Nieto et al. [12] corroborated this effect in saline media and seawater. In the case of quartz suspensions, Zhou et al. [13] found that the charge density of three cationic flocculant polymers and the NaCl concentration significantly modify the compressive yield strength, affecting the sediment structure. At a charge density of 10%, an increase in NaCl concentration increases the yield strength due to a greater compressive bridging force of the electrical double layer. In contrast, at a charge density of 100%, increasing NaCl reduces the compressive yield strength due to the reduced attraction of polymer patches. In comparison, no significant changes were observed at a charge density of 40%.

Nasser and James [14] studied the effects of the charge, density, and molecular weight of polyacrylamide on the compressibility of kaolin suspension pulps. The sediments showed greater consolidation with lower compressive yield stress in the presence of high-molecular-weight anionic polyacrylamides with low charge density. Recent studies have also shown that increasing the dosage of a high-molecular-weight flocculant, hydrolyzed polyacrylamide (HPAM), decreases the compressibility of quartz/kaolin pulps in the presence of seawater [15]. Other researchers have shown that the presence of low-molecular-weight polymers can improve consolidation, as is the case with Leong [16], who added high- and low-molecular-weight polyacrylic acid (PAA) (2000 and 750,000, respectively) to zirconium dioxide (ZrO_2_) pulp. Their results showed that low-molecular-weight PAA improved sediment consolidation because the polymer chains decreased the strength of the individual networks.

Some studies have analyzed the effects of NaPA on the rheological properties of clayey tailings concentrated in saline media [17,18]. The long and flexible chain structure allows it to wrap and surround the solid particles, which prevents them from agglomerating and settling. Robles et al. [18] analyzed the effects of NaPA on the rheological properties of kaolin pulps concentrated in seawater. The results indicated that the adsorption of the polymer on the surface of the particles improved their dispersion and significantly reduced the rheological parameters of yield stress, viscoelastic moduli, and viscosity. Jeldres et al. [17] studied the dispersion of quartz/kaolin and quartz/montmorillonite clayey tailings in seawater using NaPA. Their results indicate a decrease in the yield stress with an increase in the NaPA dosage due to steric—electrostatic repulsion of the clay particles. This is also evidenced by the negative zeta potential increase and fines shown in the particle size distribution obtained by FBRM (Focused Beam Reflectance Measurement). Recently, Ramos et al. [19] evaluated the aggregation and dispersion mechanisms of synthetic quartz/kaolin pulps in the presence of calcium and magnesium ions at pH 8 and their interactions with NaPA. All the tailings showed lower yield stress in the presence of NaPA. In addition, there was also an increase in the yield stress in the presence of calcium ions due to the hydration characteristics of this ion, which promotes stronger bonds between particles.

Colloidal stability, chemical interactions, and rheology are properties affected by the presence of Ca^2+^ and Mg^2+^ cations in clayey tailings. These cations compress the electric double layer more effectively than monovalent cations such as Na^+^, resulting in a more pronounced reduction in the zeta potential and a decrease in electrostatic repulsion between particles, promoting aggregation [20,21,22,23,24]. In seawater, Mg^2+^ tends to form Mg(OH)_2_ precipitates at alkaline pH, impairing operational performance in stages such as flotation and thickening [25,26]. Ca^2+^ cations induce greater yield stress in quartz/kaolin mineral suspensions than Mg^2+^ cations due to their lower solvation and higher adsorption on these minerals [19].

There is growing interest in exploring the performance of NaPA in modifying the rheological and compressive properties of clayey tailings in fluid media containing calcium and magnesium ions. Several investigations have addressed the compressive behavior of mineral slurries by considering variations in water hardness [11,12,13,15] and using flocculant reagents with variations in charge, molecular weight, and charge density [14,16]. In addition, the effects of dispersing reagents such as sodium pyrophosphate [27] and NaPA [17,18,19] on the rheological properties of clayey tailings in hard water have been studied.

In this context, the present research focuses on a comprehensive analysis of rheological properties, including viscoelasticity, zeta potential and compressibility, to evaluate the behavior of NaPA in clayey tailings with intensely hydrated cations such as Ca^2+^ and Mg^2+^, which act as structure-forming ions in water [28,29] and are commonly present in industrial waters. This research seeks to study the compressibility of kaolin clayey tailings in the presence of NaPA, considering the water hardness and clay composition of the tailings. The results suggest a promising scenario for optimizing water recovery in the mining industry, especially in arid regions where this water resource is scarce and seawater is used in their operations.

## 2. Materials and Methods

### 2.1. Materials

For the experiments, quartz particles (purchased from Donde Capo, Santiago, Chile) and kaolin particles (sourced from Sigma-Aldrich, St. Louis, MO, USA) were used, both with densities of 2600 kg/m^3^. High-purity NaPA, with a molecular weight of 5100 g/mol, was used as a rheology modifier and was obtained from Sigma-Aldrich (Santiago, Chile).

### 2.2. Sample Preparation

The quartz/kaolin suspensions were prepared using solutions of 0.01 M calcium chloride (CaCl_2_) and magnesium chloride (MgCl_2_). The physical and chemical properties of the solutions obtained via a HANNA H19829 multiparameter and an Anton Paar DMA35 portable density meter are shown in Table 1.

**Figure 1:**
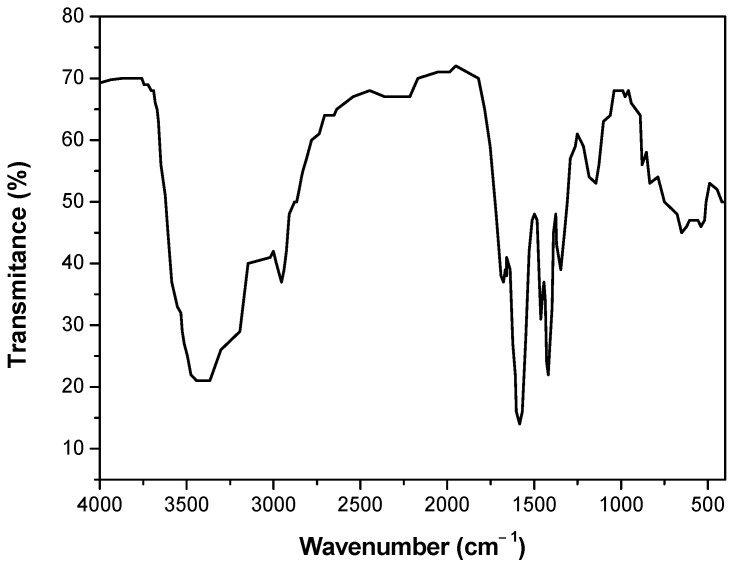
FTIR spectra of NaPA.

NaPA was characterized using Fourier transform infrared spectroscopy (FTIR) to identify its functional groups and confirm its structure before the experiments. Figure 1 shows the spectrum of NaPA obtained using a Fourier transform infrared spectrophotometer (FTIR-4600, JASCO, Tokyo, Japan) operating from 4000 to 400 cm^−1^. The wavelengths between 3500 and 3200 cm^−1^ represent the stretching vibrations of the hydroxyl group. At 1651 cm^−1^, absorption bands due to C-OH deformation vibrations can be seen. Waves between 1408 and 1563 cm^−1^ represent the asymmetric and symmetric stretching vibrations of carboxyl anions and -COO-, which refers to carboxylic acid salts. Finally, characteristic oscillation and deformation vibrations of CH_2_ are observed at 1327 and 1454 cm^−1^, respectively.

**Figure 2:**
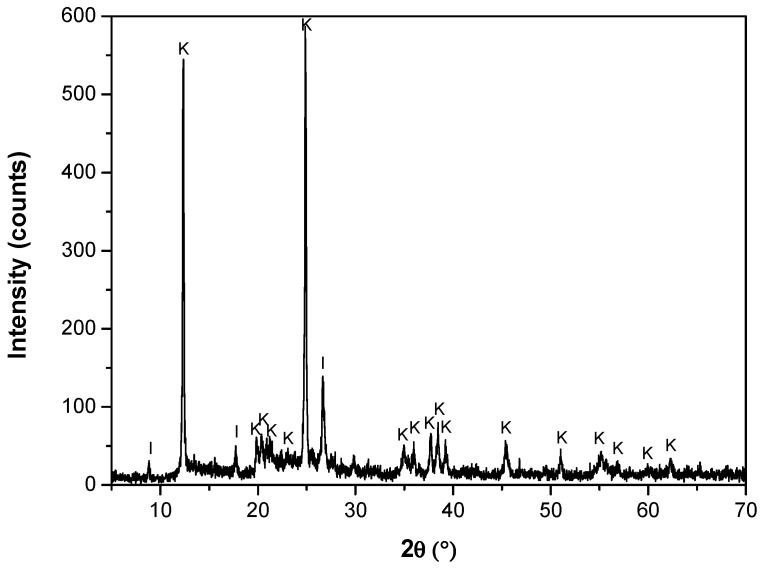
Kaolin diffractogram (I: illite, K: kaolinite).

**Figure 3:**
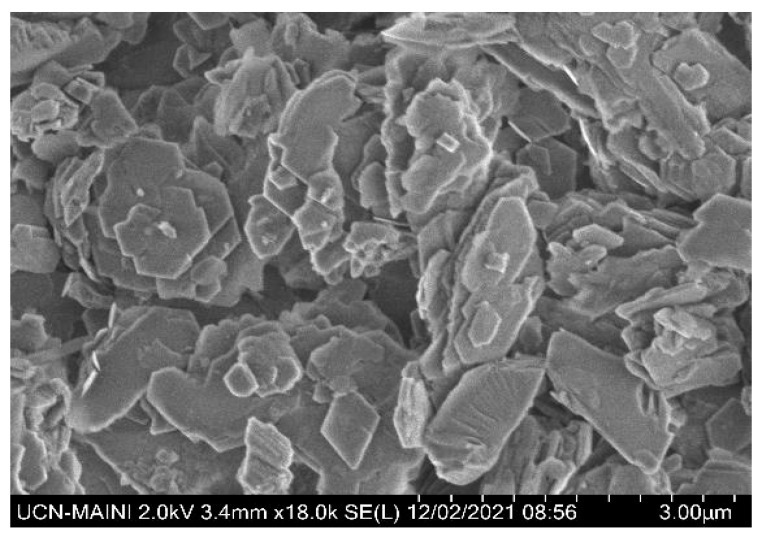
High-resolution SEM image showing surface of kaolin mineral.

The quartz (SiO_2_) content estimated by X-ray diffraction (XRD) was greater than 99% wt%, and 100% of the particles were sieved via a #212 mesh (ASTM E-11 standard [30]). The mineralogical composition of kaolin was determined using a Bruker D8 Advance X-ray diffractometer. The wavelength λ (Cukα) was 1.5406 Å. The 2θ angle studied ranged from 5° to 70°. Mineralogical components were presented using the Powder Diffraction File of ICDD (International Center for Diffraction Data). The diffractogram presented in Figure 2 confirms the presence of mostly kaolinite and illite. The scanning electron microscopy (SEM) micrograph (Figure 3) shows the presence of a population of submicron kaolin particles (<1 μm) with a hexagonal laminar crystalline structure. This analysis was performed using high-resolution SEM (Hitachi SU5000, ZRO Schottky emission electron gun) equipped with Xflash 6I30 detectors (Bruker) and a STEM detector (DEBEN). Both quartz and kaolin had a density of 2.6 kg/m^3^.

### 2.3. Methods

**Figure 4:**
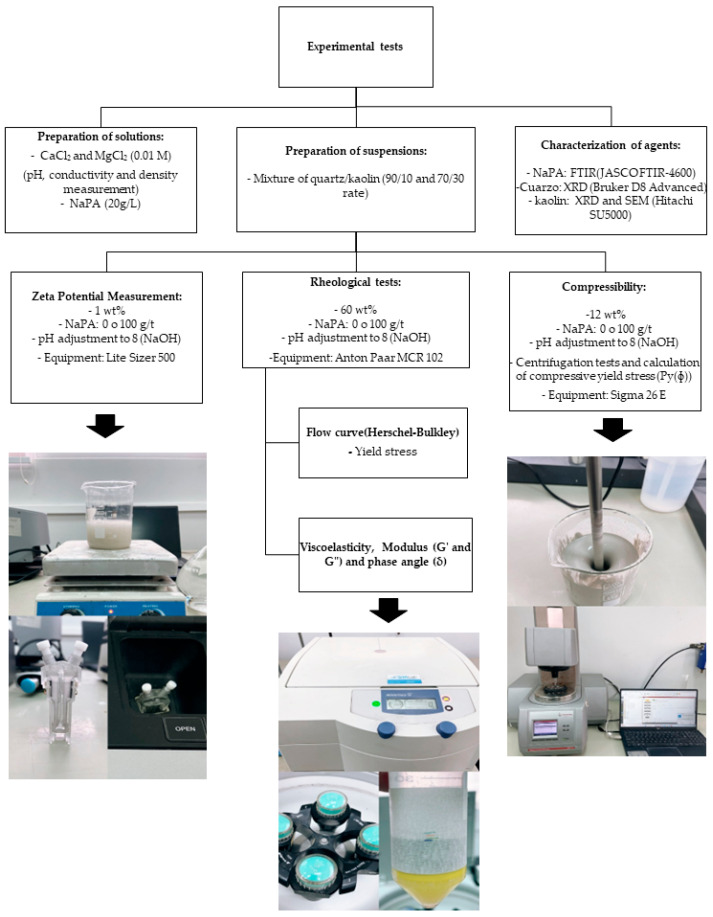
A schematic representation of the methodology.

A schematic representation of the experimental methodology is presented in Figure 4.

#### 2.3.1. Rheological Characterization

Pulp containing 150 g of quartz and kaolin mixtures in 0.01 M CaCl_2_ and MgCl_2_ solutions was prepared. The solids content was 60% by weight, while the proportions of quartz and kaolin content ranged from 70 to 90% for quartz and 10 to 30% for kaolin. NaPA was used as a dispersing agent at a 100 g/t dosage. NaOH was used as a pH modifier to achieve a pH of 8. Aliquots of 60 mL of suspension were used for each experiment. Each aliquot was used for a single test; excess aliquots were discarded. Rheological measurements were performed on an Anton Paar MCR 102 rheometer (Grupo ANAMIN, Santiago, Chile), and the data were processed using RheoCompassTM Light version software. A sandblasted #CC39 bob-in-cup configuration (1.5 mm gap) was employed to reduce wall slip effects. The initial and final shear rates were 20 and 500 s^−1^, respectively. However, the results are up to the shear rate where unsteady flows appear as Taylor vortices [31]. The sample temperature was kept constant at 25 °C. The yield stress was calculated by fitting the experimental data to the Herschel–Bulkley constitutive equation presented in Equation (1).

(1) $$\tau =\tau_{0}+kY^{n}$$

where τ is the shear stress, Y is the shear rate, $\tau_{0}$ is the yield stress, k is the consistency index, and n is the flow index.

Oscillatory rheological tests were carried out on pulps with a 60% solids content by weight. Within a linear viscoelastic regime, these tests evaluate the dependence of the storage (G’) and loss (G″) moduli on the oscillatory frequency. G’ measures the stored energy, which is related to elastic molecular events, while G″ measures the dissipated energy, which is related to viscous molecular events. The tests were performed under a frequency sweep ranging from 6.5 to 62.5 rad/s, maintaining a constant strain of 0.1%.

#### 2.3.2. Compressibility

The compressive yield stress (P_y_(Φ)) is a parameter that characterizes sediment consolidation and indicates the resistance of particles to consolidation when subjected to external stresses. P_y_(Φ) can be obtained using filtration or centrifugation techniques and provides essential information for dewatering operations related to thickening and tailings dams. This parameter explains the increase in the volumetric fraction of solids due to intercapillary water migration in the sediment pores during the described operations.

Several studies have estimated P_y_(Φ) using the batch centrifugation technique [14,15,32,33] used in the present study. Samples with a volume of 40 mL of pulp were prepared with a single volumetric solids concentration by weight of 12% (quartz/kaolin ratios of 70/30 and 90/10), maintaining the mixtures described in Section 2.1 and varying the water hardness in the absence and presence of NaPA at a dosage of 100 g/t at pH 8. The suspensions were prepared in 50 mL cylindrical tubes and transferred to a Sigma Model 26 E centrifuge with a 4-position swing-out rotor and four 50 mL cylinders. P_y_(Φ) was calculated via the method described by Green et al. [34,35], measuring the equilibrium height, $H_{eq}$, for a range of centrifugal accelerations (50–1500 $\vec{g}$). The data were then fitted to Equation (2) with a, b, and c as the best fitting parameters. The volumetric solids concentration, Φ, is obtained via Equation (3), where $\Phi_{0}$ is the initial volumetric solids concentration, $h_{0}$ is the initial height of the suspension, and R is the radius from the center of the centrifuge to the base of the test tube. The parameter P_y_(Φ) is then calculated from Equation (4), considering each selected level of centrifugal acceleration and the difference between the solid and liquid densities, ∆ρ.

(2) $$H_{eq}=a+bln\vec{g}+cln{\left(\vec{g}\right)}^{2}+dln{\left(\vec{g}\right)}^{3}\;$$

(3) $$\Phi =\frac{\Phi_{0}h_{0}\left[1-\frac{1}{2R}\left(H_{eq}+\vec{g}\frac{dH_{eq}}{d\vec{g}}\right)\right]}{\left[\left(H_{eq}+\vec{g}\frac{dH_{eq}}{d\vec{g}}\right)\left(1-\frac{H_{eq}}{R}\right)+\frac{H_{eq}^{2}}{2R}\right]}\;$$

(4) $$P_{y}\left(\Phi \right)=∆\rho \Phi_{0}h_{0}\vec{g}\left(1-\frac{H_{eq}}{2R}\right)\;$$

#### 2.3.3. Zeta Potential Measurements

Quartz and kaolin suspensions with a solids concentration of 1% by weight were prepared in solutions of CaCl_2_ and MgCl_2_. After sample homogenization, the pH was adjusted to 8 with analytical-grade sodium hydroxide. The zeta potentials of the quartz and kaolin particles were measured using a Litesizer 500 instrument (Anton Paar, Graz, Austria), which uses electrophoretic light scattering (CmPALS) as a measurement principle, using an Omega Zeta cell at 20 °C and a voltage of 220 V. All measurements were performed in triplicate for each sample to ensure reproducibility.

## 3. Results and Discussion

This section presents and analyzes the results obtained to evaluate the effects of NaPA on the physicochemical and mechanical properties of clayey tailings in the presence of different divalent cations (Ca^2+^ and Mg^2+^). The changes in zeta potential, rheological behavior (yield strength and viscoelasticity), and compressibility of the suspensions are addressed, considering different proportions of kaolin and quartz and the influence on water quality. The results are presented by test type for a clear and comparative interpretation of the effect of each variable on the system.

### 3.1. Zeta Potential

**Figure 5:**
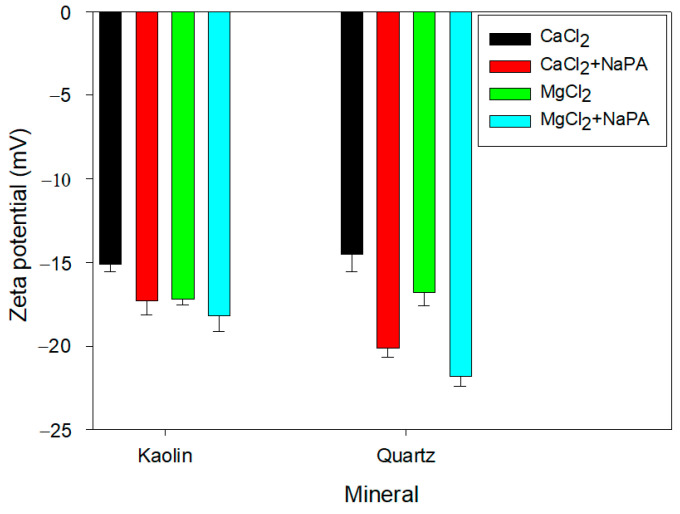
Zeta potential of kaolin and quartz suspensions in 0.01 M CaCl_2_ and MgCl_2_ solutions in the absence and presence of NaPA at pH 8.

The role of electrostatic forces in the aggregation/dispersion modes of kaolin and quartz suspensions was analyzed under the influence of different water qualities (CaCl_2_ and MgCl_2_) in the absence and presence of NaPA (100 g/t) at pH 8. This is achieved by studying the zeta potential. Figure 5 shows a more negative zeta potential in the presence of MgCl_2_ for both minerals. For cations with the same valency, such as Ca^2+^ and Mg^2+^, the difference in zeta potential is explained by the ionic hydration radius (9.6 Å for Ca and 10.8 Å for Mg). The larger the radius, the thicker and more diffuse a Stern layer generated on the kaolin and quartz minerals’ surface, which preferentially adsorbs cations with higher hydration [36,37].

The addition of NaPA increased the negative zeta potential of the particles for both water qualities due to the appearance of negative charges generated by the polymer’s carboxyl groups when ionized in aqueous solution. At pH 8, the hydroxyl ions interact with the ionized carboxyl ions of the polymer, generating a greater number of negative charges on the particle surface, which is reflected in the increased negative zeta potential.

### 3.2. Rheology

**Figure 6:**
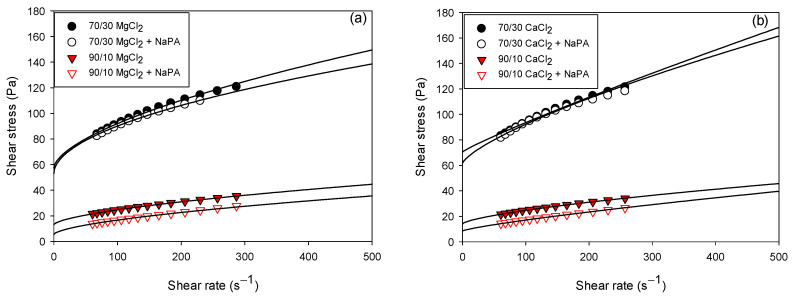
Rheograms of quartz/kaolin pulps as a function of solids content and NaPA in 0.01 M solutions of (**a**) MgCl_2_ and (**b**) CaCl_2_. The colored symbols correspond to the experimental data. The solid line represents the Herschel–Bulkley model, which is fitted to the experimental data conditions: pH 8 and a solids percentage by weight of 60%.

Figure 6 shows the rheograms of slurries with 60 wt% solids, considering the variation in quartz/kaolin ratios (70/30 and 90/10) in 0.01 M CaCl_2_ and MgCl_2_ solutions with and without NaPA at pH 8. The solid lines represent the Herschel–Bulkley values, and the colored symbols represent the experimental data. Table 2 and Table 3 show the yield strength, consistency index, and flow index values obtained with the Herschel–Bulkley model for all the tests performed. The experiments indicate that NaPA decreases the yield strength regardless of the water hardness and the quartz/kaolin component ratio due to the particle dispersion effect explained in Section 3.1.

Higher yield strengths were observed in the presence of CaCl_2_ solutions (see Table 1 and Table 2). The influence of ions on the structure of water can be understood as their impact on hydrogen bonds since they can increase or decrease the strength of these bonds. Due to their ability to induce water structuring, Ca^2+^ and Mg^2+^ ions are classified as kosmotropic ions or water structure builders. The size of the ion also plays a relevant role; larger ions such as Ca^2+^ have a greater affinity with kaolin and quartz particles and therefore tend to have low deformation [38], while Mg^2+^ ions form interparticle networks of lower resistance and are more deformable.

A greater proportion of kaolinite in the suspensions presented higher yield stress values. This behavior is attributed to the laminar morphology, particle size, and heterogeneous charges on the surface that increase the surface area of the solid particles, causing weak inter-particle networks and decreasing the rheological parameters. Several authors have analyzed the rheological behavior of clayey sludges; their results revealed pseudoplastic non-Newtonian rheological behaviors and higher rheological parameters with the increase of clayey solids in the suspension [27,39].

### 3.3. Viscoelasticity

**Figure 7:**
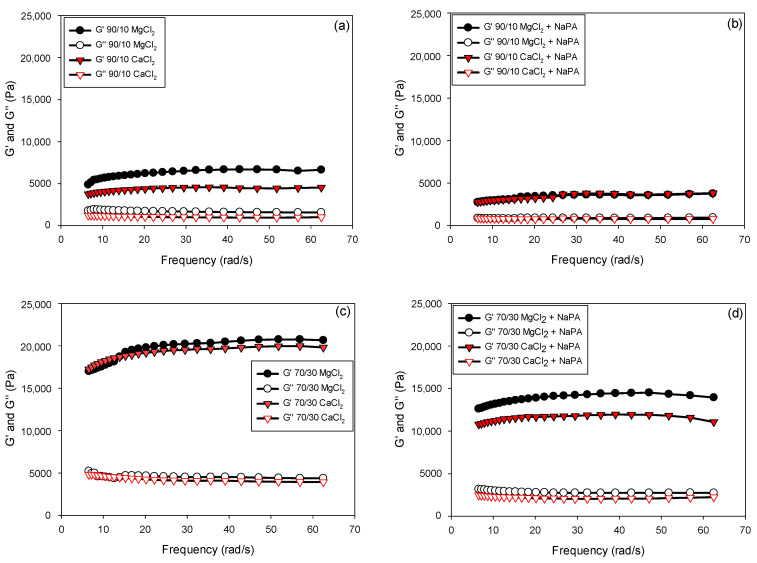
Oscillation frequency for synthetic quartz/kaolin tailings (90/10 and 70/30 ratios) in CaCl_2_ and MgCl_2_ solutions in the absence and presence of NaPA at pH 8. (**a**) 90/10, (**b**) 90/10 + NaPA, (**c**) 70/30, and (**d**) 70/30 + NaPA.

Figure 7 shows the results of the oscillatory rheological analysis performed on quartz/kaolin suspensions (90/10 and 70/30 ratios) in 0.01 M CaCl_2_ and MgCl_2_ solutions, both in the absence and presence of NaPA at pH 8. These tests were performed under frequency sweep conditions in the linear viscoelastic regime, applying a constant strain of 0.1%, as described in the methodology section.

Oscillatory analysis characterizes the mechanical behavior of the pulps under small deformations, evaluating their internal structure without significantly altering it. The results are expressed through viscoelastic moduli: G’, which represents the stored energy and is associated with elastic behavior, and G″, which reflects the energy dissipated as heat and is related to the viscous component. In all cases, G’ was greater than G″, indicating that the suspensions exhibited gel-like behavior, where elastic characteristics predominated over viscous ones.

Regarding the observed effects, the addition of NaPA (see Figure 7b,d) caused a decrease in both viscoelastic moduli, which is consistent with its dispersing action, which weakens the pulp structure. Furthermore, both salts (MgCl_2_ and CaCl_2_) exhibited higher G’ and G″ moduli with a higher proportion of kaolin. This can be attributed to the plate-shaped laminar morphology, high surface area, and water retention capacity, which favor the formation of a denser and more cohesive structural network between particles. These characteristics intensify interparticle interactions, both physically and electrostatically, resulting in suspensions with greater stiffness and elastic energy storage capacity, reflected in higher G’ and G″ values. The strength of the particles in the presence of NaPA, MgCl_2_, and CaCl_2_ solutions is represented by the phase angle (δ) between the applied stress and the strain. This can be inferred from the relationship between G″ and G’ given by Equation (5).

(5) $$\tan\;\delta =G^{\prime \prime }/G’\;$$

Low values of δ imply predominantly elastic (solid-like) behavior, while high values reflect more viscous (liquid-like) behavior. It should be noted that δ represents the ratio of G′ to G″ during oscillatory deformation and therefore does not correlate linearly with steady-state flow. However, when analyzing the effect of an additive such as a dispersant, δ can provide valuable information about which structural component of the system—elastic or viscous—is most affected.

**Figure 8:**
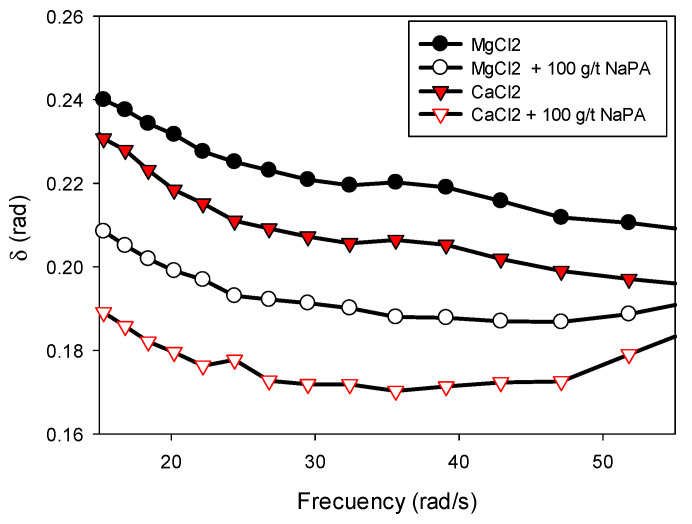
Phase angle for synthetic quartz/kaolin tailings (ratio 70/30) in CaCl_2_ and MgCl_2_ solutions in the absence and presence of NaPA at pH 8.

The results from oscillatory rheological tests (Figure 8) allow us to infer significant structural modifications in the suspension upon incorporating the NaPA dispersant. The simultaneous reduction in G′ and G″ in the presence of the additive suggests a loss of stiffness and internal cohesion of the system, attributable to the disintegration of agglomerated structures or interparticle networks previously present in the absence of additives. It is particularly noteworthy that the decrease in G″ was more pronounced than that in G′, which was reflected in a reduction in δ across the frequency range evaluated. This behavior, initially counterintuitive from a classical interpretation of δ as an indicator of “fluidity”, indicates that the dispersant impacts the system’s energy dissipation more severely than its storage mechanisms. From a structural perspective, this could be interpreted as a redistribution of interactions between particles, in which the forces acting in situations of relative motion (friction and internal collisions) are weakened more than those responsible for elastic behavior under small deformations.

In this context, the decrease in δ should not be taken as evidence of a more solid structure but rather as an indication that the system has undergone an internal reconfiguration, maintaining a relatively greater proportion of weak elastic behavior compared to viscous behavior, but on a more general basis. The suspension no longer exhibits the same structural integrity, despite the parameter δ suggesting greater “relative elasticity”. This highlights the need to interpret viscoelastic parameters in an integrated manner, considering their absolute value and joint evolution.

### 3.4. Compressibility

One of the crucial parameters for analyzing the sediment behavior of flocculated suspensions under consolidation is P_y_(Φ). This limit also provides relevant information on the variation in sediment compressibility due to variations in parameters such as particle size distribution, the presence and dosage of chemical reagents such as coagulants and flocculants, water hardness, and variations in the percentage of clays, among others. This stress provides information on the strength of the interparticle bonds when the sediment is subjected to compressive external forces. Consequently, as the value of this parameter increases, the system tends to consolidate less and retain more water. This study explores the relationship between Py(Φ) and the solid volume fraction of quartz/kaolin pulp sediment (Φ), varying the water hardness (CaCl_2_ and MgCl_2_) and the quartz/kaolin ratio (70/30 and 90/10) in the absence and presence of NaPA, following the preparation described in Section 2.3. The experiments were carried out at a solids concentration of 12% by weight and a pH of 8.

**Figure 9:**
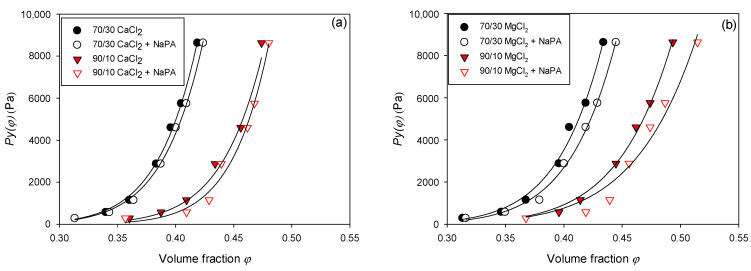
Pulp compressibility is achieved by varying the quartz/kaolin proportion and the presence of dispersants in (**a**) CaCl_2_ and (**b**) MgCl_2_.

**Figure 10:**
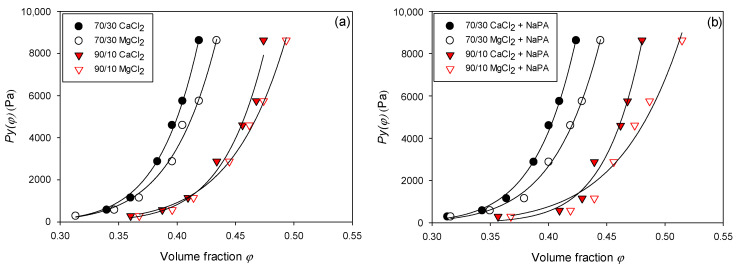
Pulp compressibility is achieved by varying the water quality and the quartz/kaolin proportion in suspension (**a**) in the absence of NaPA and (**b**) in the presence of NaPA.

The data obtained in Figure 9 and Figure 10 suggest a power law $P_{y}\left(\varphi \right)=c{\left(\varphi \right)}^{n}$, where $P_{y}\left(\varphi \right)$ is the compressive creep effort, φ is the volumetric fraction of solids, c is a constant of proportionality that indicates how low or how large it is $P_{y}\left(\varphi \right)$, and n represents the shape of the curve (the higher N, the more abrupt the change in $P_{y}\left(\varphi \right)$).

Table 4 shows the values obtained from the C and N of the quartz/kaolin suspensions with varying water qualities and quartz/kaolin proportions in the absence and presence of NaPA (0 and 100 g/t). It is observed that $P_{y}\left(\varphi \right)$ presents a strong dependence on the indicated variables. To understand the effect and impact of these variables, it is necessary to understand what this parameter depends intrinsically on and its relationship with the internal physical, microstructural, and hydraulic properties of the pulp. The compressive creep stress responds to the balance between the structural forces that support the integrity of the pulp, along with the ease/difficulty with which the liquid can escape under the action of a load or effort. In this sense, systems with high internal cohesion, low permeability, and a high degree of packaging lead to greater compressive creep.

In this context, it is observed that the capacity to consolidate the pulp prepared in both brine types (Figure 9a,b) increased in the presence of NaPA. This occurs because the reagent is a dispersant that weakens the structure of the pulp. This led to greater ease of collapse of the pulp. However, it is important to consider that reagents of this nature also reduce particle agglomeration, which could minimize the internal permeability and hydraulic connectivity between the internal pores of particle networks. However, it is not the dominant effect in this case, as it is when the proportion of clay in the system increases. This can be seen when comparing pulps with 10% vs. pulps with 30% clays. It is observed that the increase in phyllosilicates leads to greater values of compressive effort, which could be linked to the runoff capacity of water inside the pulp. Increasing the proportion of fine particles reduces the effective internal permeability, and the liquid cannot escape the system. It is also necessary to consider that clay particles have an irregular and lamellar structure, leading to a greater blockade of channels that connect internal pores, making water release more difficult.

Until now, the impact of salt on consolidation processes has only been studied by a few research groups. For example, Naser and James [11] explored the consolidation of kaolin by varying the NaCl concentration. His findings revealed that, at high salt concentrations, the sediments were more compact and had a low compressive yield stress, P_y_(Φ). This same effect was observed by Nieto et al. [12], who increased the salinity of kaolin pulps (from 0.01 m NaCl to seawater). However, studies have not yet been carried out that independently compare salts of Ca and Mg. Figure 10 shows that for both proportions of quartz/kaolin and both in the absence and presence of NAPA, the sediments show greater compactness when using MgCl_2_ instead of CaCl_2_. This can be explained by considering the size of the ions: Mg^2+^ ions have a higher hydration radius than Ca^2+^ ions.

This difference in the hydration radius between Mg^2+^ (10.8 Å) and Ca2+ (9.6 Å) directly affects the structure of the double electric layer and the interactions between particles. The Mg^2+^ ions, which are strongly hydrated, tend to stay further from the surface of the colloidal particles, generating a more diffuse layer of Stern and favoring the configurations in which weak or repulsive electrostatic interactions predominate. This allows particles to be reorganized more easily, forming more open and permeable networks that facilitate sediment consolidation and interstitial water expulsion. On the other hand, the Ca^2+^ ions, which have the lowest hydration radius, can move closer to the surface of the particles and promote ion-bridge interactions or load neutralization, which leads to dense, rigid, and less deformable structural networks. This greater internal cohesion hinders water migration through sediment during compression, resulting in a greater Py(Φ). Therefore, the type of ion modifies the superficial affinity and regulates the microstructure of the sediment and its mechanical response against external efforts.

## 4. Conclusions

The effects of NaPA on the rheological and compressive properties of clay tailings were studied by analyzing different concentrations of kaolin and water hardness (CaCl_2_ and MgCl_2_). The following conclusions are drawn:

NAPA acted as an efficient rheological modifier for clay tailings in hard water, significantly reducing the rheological parameters (creep stress and viscoelastic moduli) and improving the sediment’s compressibility. This effect is attributed to its highly ionizable anionic structure, which generates steric and electrostatic repulsion between particles, promoting dispersed and easily consolidable structures. The yield stress decreased from 14.12 to 8.57 Pa in CaCl_2_ and 13.70 to 5.45 Pa in MgCl_2_ for the quartz/kaolin 90/10 mixture. For the quartz/kaolin 70/30 mixture, the yield stress decreased from 70.4 to 61.64 Pa in CaCl_2_ and from 57.51 to 52.95 Pa in MgCl_2_.

Water quality, particularly divalent cations such as Ca^2+^ and Mg^2+^, determines mineral pulps’ rheological and compressive behavior. The suspensions with Mg^2+^ showed greater compressibility than those with Ca^2+^ due to their highest hydration radius, which originates from networks of weaker and permeable particles, facilitating water release.

The proportion of kaolin in the mixture directly affects the system’s response, observing that a greater fraction of kaolin increases the creep effort and reduces the compressibility of the sediment. This effect is related to the laminar morphology, colloidal size, and high surface area of kaolin, which generate more compact structures and deformation resistance.

Zeta potential measurements showed that adding NaPA significantly increased the negative charge on the surface of the quartz and kaolin particles in both the CaCl_2_ and MgCl_2_ solutions. Specifically, in the presence of MgCl_2_, the zeta potential shifted from −10.7 mV to −19.2 mV for quartz and from −8.6 mV to −16.3 mV for kaolin. This increase in negative zeta potential reflects enhanced electrostatic repulsion between particles, promoting better dispersion and reduced aggregation.

## Figures and Tables

**Table 1:** Chemical and physical properties of CaCl_2_ and MgCl_2_ solutions at 23 °C, 1 atm, and 0.01 M.

Solution	CaCl_2_	MgCl_2_
pH	6.5	6.0
Density (kg/m^3^)	995.8	997.4
Electric conductivity (μS)	2015	2202

**Table 2:** Herschel–Bulkley parameters for quartz/kaolin suspensions in 0.01 M MgCl_2_ in the absence and presence of NaPA at pH 8.

Quartz/Kaolin	Yield Stress [Pa]	Consistency Index	Flow Index
70/30	57.51	2.09	0.61
70/30 + NaPA	52.95	3.42	0.52
90/10	13.07	0.66	0.62
90/10 + NaPA	5.45	0.67	061

**Table 3:** Herschel–Bulkley parameters for quartz/kaolin suspensions in 0.01 M CaCl_2_ in the absence and presence of NaPA at pH 8.

Quartz/Kaolin	Yield Stress [Pa]	Consistency Index	Flow Index
70/30	70.54	0.37	0.89
70/30 + NaPA	61.64	1.04	0.73
90/10	14.12	0.46	0.68
90/10 + NaPA	8.57	0.21	0.81

**Table 4:** Compressive yield stress and power law parameters for quartz/kaolin suspensions (proportions 70/30 and 90/10), varying the water quality, and in the presence and absence of NaPA.

Quartz/Kaolin	Brine	0 g/t NaPA			100 g/t NaPA		
		*c*(*x*10^7^)*Pa*	*n*	*R* ^2^	*c*(*x*10^7^)*Pa*	*n*	*R* ^2^
70/30	CaCl_2_	34.50	12.19	0.99	20.50	11.77	0.99
	MgCl_2_	7.78	10.95	0.99	3.57	10.36	0.99
90/10	CaCl_2_	7.30	12.28	0.97	4.61	12.00	0.98
	MgCl_2_	4.52	12.01	0.99	1.54	11.17	0.96

## Data Availability

The original contributions presented in this study are included in the article. Further inquiries can be directed to the corresponding author.

## Glossary of Symbols

τ	Shear stress (Pa)
$\tau_{0}$	Yield stress (Pa)
k	Consistency index
Y	Shear rate (s^−1^)
n	Flow index
G’	Elastic modulus (Pa)
G″	Viscoelastic modulus (Pa)
$P_{y}\left(\Phi \right)$	Compressive yield stress (Pa)
Φ	Volume fraction of solids
$\vec{g}$	Centrifugal acceleration (m/s^2^)
$H_{eq}$	Equilibrium height (m)
$\Phi_{0}$	Initial volumetric solids concentration
$h_{0}$	Initial height of the suspension (m)
∆ρ	Difference between the solid and liquid densities (Kg/m^3^)
δ	Phase angle (rad)

## References

[1] Cisternas L.A., Gálvez E.D. (2018). The Use of Seawater in Mining. Miner. Process. Extr. Metall. Rev..

[2] de Kretser R., Scales P.J., Boger D.V. (1997). Improving Clay-Based Tailings Disposal: Case Study on Coal Tailings. AIChE J..

[3] Arjmand R., Massinaei M., Behnamfard A. (2019). Improving Flocculation and Dewatering Performance of Iron Tailings Thickeners. J. Water Process Eng..

[4] Shadrunova I.V., Gorlova O.E., Galyamov V.S. (2018). Use of Rheology Modifiers to Adapt Storage of Tailings of Enrichment. IOP Conf. Ser. Mater. Sci. Eng..

[5] Bustos M.C., Concha F., Bürger R., Tory E.M. (1999). Sedimentation and Thickening. Phenomenological Foundation and Mathematical Theory.

[6] Green M.D. (1997). Characterisation of Suspensions in Settling and Compresion.

[7] Lester D.R., Usher S.P., Scales P.J. (2005). Estimation of the Hindered Settling Function R(φ) from Batch-Settling Tests. AIChE J..

[8] Channell G.M., Miller K.T., Zukoski C.F. (2000). Effects of Microstructure on the Compressive Yield Stress. AIChE J..

[9] Buscall R. (1982). The Elastic Properties of Structured Dispersions: A Simple Centrifuge Method of Examination. Colloids and Surfaces.

[10] Buscall R., White L.R. (1987). The Consolidation of Concentrated Suspensions. Part 1.—The Theory of Sedimentation. J. Chem. Soc. Faraday Trans. 1 Phys. Chem. Condens. Phases.

[11] Nasser M.S., James A.E. (2006). Settling and Sediment Bed Behaviour of Kaolinite in Aqueous Media. Sep. Purif. Technol..

[12] Nieto S., Piceros E., Toledo P.G., Robles P., Jeldres R. (2023). Compressive Yield Stress of Flocculated Kaolin Suspensions in Seawater. Polymers.

[13] Zhou Y., Jameson G.J., Franks G.V. (2008). Influence of Polymer Charge on the Compressive Yield Stress of Silica Aggregated with Adsorbed Cationic Polymers. Colloids Surfaces A Physicochem. Eng. Asp..

[14] Nasser M.S., James A.E. (2006). The Effect of Polyacrylamide Charge Density and Molecular Weight on the Flocculation and Sedimentation Behaviour of Kaolinite Suspensions. Sep. Purif. Technol..

[15] Nieto S., Toledo P.G., Robles P., Quezada G.R., Jeldres R.I. (2023). Impact of Magnesium on the Flocculation, Sedimentation and Consolidation of Clay-Rich Tailings in Lime-Treated Seawater. Sep. Purif. Technol..

[16] Leong Y.K. (1994). Exploitation of Interparticle Forces in the Processing of Colloidal Ceramic Materials. Mater. Des..

[17] Jeldres M., Robles P., Toledo P.G., Saldaña M., Quezada L., Jeldres R.I. (2021). Improved Dispersion of Clay-Rich Tailings in Seawater Using Sodium Polyacrylate. Colloids Surfaces A Physicochem. Eng. Asp..

[18] Robles P., Piceros E., Leiva W.H., Valenzuela J., Toro N., Jeldres R.I. (2019). Analysis of Sodium Polyacrylate as a Rheological Modifier for Kaolin Suspensions in Seawater. Appl. Clay Sci..

[19] Ramos J.J., Nieto S., Quezada G.R., Leiva W., Robles P., Betancourt F., Jeldres R.I. (2024). Rheological Behavior of Clay Tailings in the Presence of Divalent Cations and Sodium Polyacrylate: Insights from Molecular Dynamics Simulations. Polymers.

[20] Jeldres R.I., Piceros E.C., Leiva W.H., Toledo P.G., Herrera N. (2017). Viscoelasticity and Yielding Properties of Flocculated Kaolinite Sediments in Saline Water. Colloids Surfaces A Physicochem. Eng. Asp..

[21] Basnayaka L., Subasinghe N., Albijanic B. (2017). Influence of Clays on the Slurry Rheology and Flotation of a Pyritic Gold Ore. Appl. Clay Sci..

[22] Forbes E., Chryss A. (2017). Fundamentals of Clays: Surface and Colloid Science, and Rheology. Clays in the Minerals Processing Value Chain.

[23] Avadiar L., Leong Y.-K., Fourie A., Nugraha T., Clode P.L. (2014). Source of Unimin Kaolin Rheological Variation–Ca^2+^ Concentration. Colloids Surfaces A Physicochem. Eng. Asp..

[24] Avadiar L., Leong Y.K., Fourie A. (2015). Physicochemical Behaviors of Kaolin Slurries with and without Cations-Contributions of Alumina and Silica Sheets. Colloids Surfaces A Physicochem. Eng. Asp..

[25] Castro S., Lopez-Valdivieso A., Laskowski J.S. (2016). Review of the Flotation of Molybdenite. Part I: Surface Properties and Floatability. Int. J. Miner. Process..

[26] Ramos J.J., Leiva W.H., Castillo C.N., Ihle C.F., Fawell P.D., Jeldres R.I. (2020). Seawater Flocculation of Clay-Based Mining Tailings: Impact of Calcium and Magnesium Precipitation. Miner. Eng..

[27] Leiva W., Ayala L., Robles P., Nieto S., Castellón C., Herrera N., Jeldres R. (2024). Sodium Acid Pyrophosphate as a Rheological Modifier of Clay-Based Tailings in Saline Water. Appl. Clay Sci..

[28] Hribar B., Southall N.T., Vlachy V., Dill K.A. (2002). How Ions Affect the Structure of Water. J. Am. Chem. Soc..

[29] Gun’ko V.M., Andriyko L.S., Zarko V.I., Marynin A.I., Olishevskyi V.V., Janusz W. (2014). Effects of Dissolved Metal Chlorides on the Behavior of Silica Nanoparticles in Aqueous Media. Cent. Eur. J. Chem..

[30] (2023). Standard Specification for Woven Wire Test Sieve Cloth and Test Sieves.

[31] Taylor G. (1923). VIII. Stability of a Viscous Liquid Contained between Two Rotating Cylinders. Philos. Trans. R. Soc. London. Ser. A.

[32] Nasser M.S., James A.E. (2008). Compressive and Shear Properties of Flocculated Kaolinite–Polyacrylamide Suspensions. Colloids Surfaces A Physicochem. Eng. Asp..

[33] Nasser M.S., James A.E. (2007). Effect of Polyacrylamide Polymers on Floc Size and Rheological Behaviour of Kaolinite Suspensions. Colloids Surfaces A Physicochem. Eng. Asp..

[34] Green M.D., Eberl M., Landman K.A. (1996). Compressive Yield Stress of Flocculated Suspensions: Determination via Experiment. AIChE J..

[35] Green M.D., Boger D.V. (1997). Yielding of Suspensions in Compression. Ind. Eng. Chem. Res..

[36] Mitchell J.K., Soga K. (2005). Soil–Water–Chemical Interactions.

[37] Yukselen Y., Kaya A. (2003). Zeta Potential of Kaolinite in the Presence of Alkali, Alkaline Earth and Hydrolyzable Metal Ions. Water. Air. Soil Pollut..

[38] Jeldres R.I., Piceros E.C., Leiva W.H., Toledo P.G., Quezada G.R., Robles P.A., Valenzuela J. (2019). Analysis of Silica Pulp Viscoelasticity in Saline Media: The Effect of Cation Size. Minerals.

[39] Cruz N., Peng Y., Wightman E., Xu N. (2015). The Interaction of Clay Minerals with Gypsum and Its Effects on Copper–Gold Flotation. Miner. Eng..

